# Frequent *MAGE* Mutations in Human Melanoma

**DOI:** 10.1371/journal.pone.0012773

**Published:** 2010-09-16

**Authors:** Otavia L. Caballero, Qi Zhao, Donata Rimoldi, Brian J. Stevenson, Suzanne Svobodová, Sylvie Devalle, Ute F. Röhrig, Anna Pagotto, Olivier Michielin, Daniel Speiser, Jedd D. Wolchok, Cailian Liu, Tanja Pejovic, Kunle Odunsi, Francis Brasseur, Benoit J. Van den Eynde, Lloyd J. Old, Xin Lu, Jonathan Cebon, Robert L. Strausberg, Andrew J. Simpson

**Affiliations:** 1 Ludwig Institute for Cancer Research Ltd, New York Branch at Memorial Sloan-Kettering Cancer Center, New York, New York, United States of America; 2 J. Craig Venter Institute, Rockville, Maryland, United States of America; 3 Ludwig Institute for Cancer Research Ltd, Lausanne Branch, Lausanne, Switzerland; 4 Ludwig Institute for Cancer Research Ltd, Melbourne Centre for Clinical Sciences, Austin Health, Heidelberg, Victoria, Australia; 5 Nuffield Department of Clinical Medicine, University of Oxford, Ludwig Institute for Cancer Research Ltd, Oxford Branch, Headington, Oxford, United Kingdom; 6 Department of Medicine and Ludwig Center for Cancer Immunotherapy, Memorial Sloan-Kettering Cancer Center, New York, New York, United States of America; 7 Division of Gynecologic Oncology and the Knight Cancer Institute, Oregon Health & Science University, Portland, Oregon, United States of America; 8 Department of Gynecological Oncology and Center for Immunotherapy, Roswell Park Cancer Institute, Buffalo, New York, United States of America; 9 Ludwig Institute for Cancer Research Ltd, Brussels Branch, Université catholique de Louvain, Brussels, Belgium; 10 Ludwig Institute for Cancer Research Ltd, New York, New York, United States of America; Duke-National University of Singapore Graduate Medical School, Singapore

## Abstract

**Background:**

Cancer/testis (CT) genes are expressed only in the germ line and certain tumors and are most frequently located on the X-chromosome (the CT-X genes). Amongst the best studied CT-X genes are those encoding several MAGE protein families. The function of MAGE proteins is not well understood, but several have been shown to potentially influence the tumorigenic phenotype.

**Methodology/Principal Findings:**

We undertook a mutational analysis of coding regions of four CT-X *MAGE* genes, *MAGEA1*, *MAGEA4*, *MAGEC1*, *MAGEC2* and the ubiquitously expressed *MAGEE1* in human melanoma samples. We first examined cell lines established from tumors and matching blood samples from 27 melanoma patients. We found that melanoma cell lines from 37% of patients contained at least one mutated *MAGE* gene. The frequency of mutations in the coding regions of individual *MAGE* genes varied from 3.7% for *MAGEA1* and *MAGEA4* to 14.8% for *MAGEC2*. We also examined 111 fresh melanoma samples collected from 86 patients. In this case, samples from 32% of the patients exhibited mutations in one or more *MAGE* genes with the frequency of mutations in individual *MAGE* genes ranging from 6% in *MAGEA1* to 16% in *MAGEC1*.

**Significance:**

These results demonstrate for the first time that the *MAGE* gene family is frequently mutated in melanoma.

## Introduction

Cancer/testis (CT) genes are expressed primarily in the germ line but are also active in a number of human tumors including those of the lung, breast, ovary and skin [1]. Amongst the CT-genes is a subset with very tight transcriptional regulation that is specifically expressed in spermatogonia, completely undetectable in somatic tissues and encoded on the X-chromosome [2]. The proteins derived from these CT-X genes are significantly immunogenic when aberrantly expressed in human tumors and are being widely studied in the context of therapeutic cancer vaccines [3], [4]. Currently, two phase III trials are being undertaken with a vaccine containing the CT-X protein MAGEA3 as an adjuvant therapy for non-small cell lung cancer and melanoma [5].

Due to their strong up regulation in tumors, it has been widely speculated that the CT-X genes might play a role in the tumorigenic process. This has been difficult to prove, however, as their function remains obscure. Nevertheless a number of *in vitro* studies, focused on the MAGE proteins, have found evidence that they can interfere with p53 mediated apoptosis and promote cell proliferation [6], [7], [8], [9], [10]. In addition, a number of studies have found CT-X expression to be linked with both more advanced and more aggressive tumors [11], [12], [13]. To complicate this scenario, however, there have also been observations that link the expression of individual MAGE genes with a better prognosis and longer survival [14], [15], [16].

Recently, it has begun to be possible to undertake genome-wide investigations of somatic mutations in human tumors [17], [18], [19]. Within the published data, we identified reports of missense mutations in the CT-X antigen genes *MAGEA1*, *MAGEA4*, *MAGEC1*, *MAGEC2*, as well as the genes for the ubiquitously expressed *MAGEE1* (also encoded on the X chromosome) in breast and brain tumors [17], [18]. Although the frequency of these mutations is low, we reasoned that their mutation might not be simply due to chance as none were observed to be mutated in colon or pancreatic tumors although the same genes were sequenced in similar numbers of tumors [17], [19]. Furthermore, *MAGEE1* was mutated sufficiently frequently to be classified as a candidate cancer gene (CAN-gene) in breast cancer and thus potentially a driver of tumorigenesis [17].

Since greater knowledge of somatic *MAGE* mutations in human tumors might cast further light on their potential role in tumorigenesis, as well as provide important information relevant to the use of MAGE proteins in cancer vaccines, we have undertaken a systematic mutational analysis of the coding regions of the five MAGE genes in which mutations were reported (*MAGEA1, MAGEA4*, *MAGEC1*, *MAGEC2*, *MAGEE1)*. For this study we used human melanoma and ovarian samples, two tumor types with frequent CT-X expression. Our results reveal that one or more of these genes is mutated in around 35% of melanomas with some tumors exhibiting multiple mutations in these genes. On the other hand we found no mutations of these genes in ovarian tumors. Further investigations will be required to determine whether these mutations are drivers or passengers of tumorigenesis.

## Materials and Methods

### Sequence analysis of the melanoma samples

Tumor and matching blood samples from 27 melanoma cancer patients were collected at Lausanne University Hospital (CHUV), Switzerland. Cell lines were established from these samples at the Ludwig Institute of Cancer Research, from fresh surgery samples using mechanical or a combination of mechanical and enzymatic dissociation. All cell lines were derived from cutaneous melanomas, except for T1257A and B (mucosal melanoma). They were all from tumor metastases, except for LAU-Me300 and LAU-T1257A, which were from primary tumors. The following pairs of cell lines were established from the same patients: LAU-Me260.LN and LAU-T149D (patient 149, 7 years apart); LAU-Me275 and LAU-T50B (patient 50, 12 years apart); LAU-Me 261 and LAU-T42B (patient 42, 3 years apart); LAU-Me305 and LAU-Me317.M2 (patient 233, 6 months apart); LAU-T1257A and C (patient 1257, primary tumor and synchronous metastasis, respectively); LAU-T1262 A and B (patient 1262, synchronous metastases); LAU-T1255A/B are two independent lines from a large tumor. Established cultures were confirmed to be from human melanoma by flow cytometric analysis with antibodies against the high molecular weight melanoma- associated antigen and MHC class I molecules. Additional phenotyping was performed by flow cytometry, Western blotting and RT/PCR to assess expression of melanoma/melanocytic antigens (e.g. MART-1, tyrosinase, cancer/testis genes). Cell lines were routinely tested and found negative for mycoplasma. Cells were periodically checked for morphology and expression of selected antigens by RT/PCR.

In addition, fresh tumor and blood samples were collected from 86 patients attending the Melanoma Clinic at Austin Health, Melbourne, Australia (Table S2). A written informed consent was obtained from all participating subjects. This study was approved by the Ethics Committee for Clinical Research from the University of Lausanne, Switzerland, by the Human Research Ethics Committee, Research Ethics Unit, Austin Hospital, Australia and by the Ethics Committee, J. Craig Venter Institute.

Genomic DNA was extracted using a Qiagen kit following a standard protocol. Targeted sequencing was carried out with a fully automated and high-throughput production pipeline that is based on polymerase chain reaction (PCR) amplification of genomic DNA followed by traditional Sanger sequencing chemistry as previously described [20]. Primer sequences are listed in Table S3. Mutational analysis was done by comparing the sequence traces between tumors and their matching blood samples. Each somatic mutation call had to be supported by both forward and reverse traces of each amplicon and was manually verified.

### Cloning of and sequencing analysis of mutated *MAGEA1* in LAU-Me190

PCR was undertaken with High Fidelity Taq polymerase (Invitrogen, Carlsbad, CA) plus 10 pmol of each of the following primers in 25 µl to amplify the region containing the sequence variation in the tumor corresponding to the LAU-Me190 cell line: Forward 5′-AGAAAACCAACCAAATCAGCCA-3′and Reverse 5′-TCATGTCTCTTGAGCAGAGGAGTCT-3′. The amplification consisted of 35 cycles of a denaturation step at 94°C for 30 s, followed by 30 s at 55°C and extension at 68°C for 30 s followed by a final 7-min extension. PCR products were loaded onto 1.5% agarose gel, stained with ethidium bromide and visualized by UV illumination. The predicted size of the MAGEA1 PCR product was 340 bp. The PCR product was recovered and purified after agarose gel electrophoresis using a QIAquick Gel Extraction Kit (Qiagen, Valencia, CA). Cloning in pcDNA™3.1/V5-His was performed at room temperature for 30 minutes in a total volume of 6 µl using the pcDNA™3.1/V5-His TOPO® TA Expression Kit (Invitrogen, Carlsbad, CA). Transformation was performed into chemically competent One Shot® TOP10 E. coli (Invitrogen, Carlsbad, CA) that were plated in LB plates containing 100 µg/ml ampicillin and incubated overnight at 37°C. Fifteen colonies were picked and grown overnight in LB medium containing 100 µg/ml ampicillin. Plasmids were isolated with Wizard® Plus SV Minipreps DNA Purification System (Promega Madison, WI). DNA was submitted to Sanger sequencing using the T7 promoter primer.

## Results and Discussion

The entirety of *MAGEA1*, *MAGEA4* and *MAGEC2* genes and at least 70% of *MAGEC1* and *MAGEE1* could be specifically PCR amplified thus permitting detection of somatic mutations by conventional Sanger sequencing. Some regions of *MAGEC1* and *MAGEE1* could not be covered by PCR amplicons due to very high GC content or repetitive sequences.

To avoid the complication of contaminating normal tissues in fresh tumor samples, we first undertook a Discovery Screen for *MAGE* mutations using melanoma cell lines and corresponding EBV transformed leukocytes from 27 patients treated at the University Hospital in Lausanne, Switzerland (CHUV). We detected a total of 15 somatic *MAGE* coding region mutations in these cell lines, with at least one mutation in each of the five genes examined (Table 1). Two other genes were also sequenced in these samples, *PRAME*, on chromosome 22 and *DDX53* on the X-chromosome, and served as negative controls as no mutations were found. As a positive control, the expected mutation frequencies of the major cancer genes *TP53* and *BRAF* were found in these cell lines (Table S1).

**Table 1:** Somatic mutations identified in MAGE genes in the discovery set.

Gene	Sample name	Amino acid	Codon change	TP53 status	BRAF status
MAGEA1	LAU-Me190	S33F	TCC>TTC	WT	V600E
MAGEA1	LAU-Me190	L129L	CTG>CTA	WT	V600E
MAGEA4	LAU-Me190	E34K	GAG>AAG	WT	V600E
MAGEC1	LAU-Me190	E877K	GAG>AAG	WT	V600E
MAGEC1	LAU-Me190	F144F	TTC>TTT	WT	V600E
MAGEC1	LAU-Me243	P756S	CCC>TCC	WT	WT
MAGEC1	LAU-Me200	G769R	GGG>AGG	S241F	WT
MAGEC2	LAU-Me275	S110N	AGC>AAC	WT	V600E
MAGEC2	LAU-Me243	R271R	AGG>AGA	WT	WT
MAGEC2	LAU-Me300	S111L	TCA>TTA	WT	V600E
MAGEC2	LAU-Me280.R.LN	P295L	CCA>CTA	P278S	G593S; L597R
MAGEC2	LAU-Me261	S51F	TCC>TTC	S241F	WT
MAGEE1	LAU-Me290	P324S	CCT>TCT	WT	WT
MAGEE1	LAU-T441A	P568L	CCC>CTC	WT	WT
MAGEE1	LAU-Me281	T330T	ACC>ACG	WT	V600E

Overall, cell lines from 10 of the 27 patients exhibited *MAGE* mutations (37%). From five of the patients where MAGE mutations were found, fresh tumor tissue was also available. In four of these we were also able to identify the mutation found in the cell line in the fresh tissue. The exception was the tumor matching LAU-Me190 cells (five different mutations were found in the latter). One explanation for this could be tumor heterogeneity, which would render mutations present in small subset of cells undetectable by the sequencing technology used. To investigate this possibility, we selected one mutation, S33F in MAGEA1, for further study. We amplified the mutated region from the tumor tissue and cloned the amplification products into a plasmid and sequenced the clones. Two of 15 clones were found to contain the mutation. Thus for this mutation we were able to confirm the mutation in the original tissue in a subset of alleles.

Two of the mutations detected occurred in cell lines from patients for which additional autologous melanoma lines established from separate metastases were available. In one case, the lines (LAU-Me275 and LAU-T50B) were established twelve years apart and in the second (cell lines LAU-Me261 and LAU-T42B), the lines were established three years apart. We tested these additional lines for the presence of the mutations. In both cases, the mutations were found in the paired asynchronous cell lines.

Based on our finding of *MAGE* mutations in the melanoma cell line samples, a Validation Screen was undertaken in which we sequenced the same *MAGE* genes in 111 fresh tumor samples collected from 86 melanoma patients who had undergone surgical intervention at the Melanoma Clinic at Austin Health, Melbourne, Australia (Table 2). In addition, we sequenced the same genes in 33 samples from ovarian tumors collected at Roswell Park Cancer Center, Buffalo, New York. We identified additional somatic coding region mutations for each of the genes in the melanoma samples. Overall, 32% of the melanoma patients had a mutation in at least one of the genes sequenced. The frequencies of patients with missense or nonsense mutations for the individual genes were 5.8%, 11.6%, 15.1% and 7.0% and 7.0% for *MAGEA1*, *MAGEA4*, *MAGEC1*, *MAGEC2* and *MAGEE1* respectively. More than one sample was available from six of the patients. In all cases where a *MAGE* mutation was found in one sample, it was also found to be present in the other samples from the same patient. We observed coding region mutation frequencies of 47.8% and 20.4% respectively for *BRAF* and *TP53* in the samples in the Validation Screen (Table S2). These frequencies are both consistent with those reported by others for these genes in melanoma arguing that our findings are representative. In contrast to the melanoma samples, no *MAGE* mutations were identified in any of the ovarian samples sequenced although a mutation frequency of 31% was found for *TP53* (Table S4). This frequency is consistent with the findings of others [21] suggesting that the samples were sufficiently enriched for tumor derived cells to permit the detection of *MAGE* mutations were they to be present. The apparent lack of mutations in the ovarian samples studied points to a distinct tumorigenic pathway for the ovarian tumors and melanoma where MAGE genes may play different roles.

**Table 2:** Somatic mutations identified in MAGE genes in the validation set.

Gene	Sample name	Amino acid	Codon change	TP53 status	BRAF status
MAGEA1	04-007	S296P	TCC>CCC	WT	V600E
MAGEA1	7552	L271F	CTC>TTC	WT	V600E
MAGEA1	4198	E217K	GAG>AAG	R110C, Q100stop, P36L	NA
MAGEA1	6227	D258A	GAT>GCT	FS at H179*	WT
MAGEA1	6613	R236K	AGG>AAG	R181P	WT
MAGEA4	6541	G316R	GGA>AGA	WT	V600E
MAGEA4	7889	S99L	TCG>TTG	R282P	WT
MAGEA4	4198	E138K	GAG>AAG	R110C, Q100stop, P36L	NA
MAGEA4	4198	P149S	CCT>TCT	R110C, Q100stop, P36L	NA
MAGEA4	6985	E224K	GAG>AAG	WT	WT
MAGEA4	02-105	P267S	CCT>TCT	WT	WT
MAGEA4	7194	E242K	GAG>AAG	R196stop	WT
MAGEA4	03-043	R269C	CGC>TGC	WT	WT
MAGEA4	5668	I222I	ATC>ATT	WT	WT
MAGEA4	2112	E21E	GAG>GAA	WT	V600E
MAGEA4	5558	P45S	CCT>TCT	S241F	WT
MAGEC1	07-223	F904F	TTC>TTT	WT	V600E
MAGEC1	04-007	P26L	CCT>CTT	WT	V600E
MAGEC1	04-007	L35F	CTC>TTC	WT	V600E
MAGEC1	7889	E991K	GAG>AAG	R282P	WT
MAGEC1	4198	E59E	GAG>GAA	R110C, Q100stop, P36L	NA
MAGEC1	4198	D62N	GAC>AAC	R110C, Q100stop, P36L	NA
MAGEC1	4198	P38S	CCC>TCC	R110C, Q100stop, P36L	NA
MAGEC1	6985	P83S	CCC>TCC	WT	WT
MAGEC1	6985	S688F	TCC>TTC	WT	WT
MAGEC1	6985	S863L	TCA>TTA	WT	WT
MAGEC1	6458	K1104K	AAG>AAA	R248W	WT
MAGEC1	7151	P119S	CCT>TCT	WT	V600E
MAGEC1	7194	Q664stop	CAG>TAG	R196stop	WT
MAGEC1	7194	E668E	GAG>GAA	R196stop	WT
MAGEC1	7194	P127L	CCT>CTT	R196stop	WT
MAGEC1	4066	F904F	TTC>TTT	WT	V600E
MAGEC1	02-024	L705L	CTG>TTG	R213stop	WT
MAGEC1	6795	G986E	GGG>GAG	WT	WT
MAGEC1	4985	S964S	TCC>TCA	WT	WT
MAGEC1	4985	S18S	TCC>TCT	WT	WT
MAGEC1	4985	D687N	GAT>AAT	WT	WT
MAGEC1	4062	P50S	CCT>TCT	WT	V600K
MAGEC1	03-063	S134S	TCC>TCT	WT	G596R
MAGEC2	07-223	P3S	CCC>TCC	WT	V600E
MAGEC2	4198	F151Y	TTC>TAC	R110C, Q100stop, P36L	NA
MAGEC2	4198	E36E	GAG>GAA	R110C, Q100stop, P36L	NA
MAGEC2	6985	S58F	TCC>TTC	WT	WT
MAGEC2	7516	F265F	TTC>TTT	R337S	WT
MAGEC2	7259	D335N	GAT>AAT	WT	WT
MAGEC2	02-102	P84P	CCC>CCT	WT	V600E
MAGEE1	6541	D446C	GAT>TGT*	WT	V600E
MAGEE1	4198	R711K	AGG>AAG	R110C, Q100stop, P36L	NA
MAGEE1	6985	S319S	TCC>TCT	WT	WT
MAGEE1	03-091	A717V	GCT>GTT	WT	G469E
MAGEE1	08-249E	A859A	GCC>GCT	WT	V600K
MAGEE1	8022	S67F	TCC>TTC	WT	V600E
MAGEE1	4985	E692D	GAA>GAT	WT	WT
MAGEE1	4985	E693K	GAA>AAA	WT	WT

In addition to generating new mutation data, we also combed the publically available databases and found a small number of additional mutations in lung tumors, glioblastoma, breast tumors and melanoma in *MAGE* genes [17], [18], [19], [22] (Table 3). On the other hand, some tumor types known to express CT-X genes, such as colon, bladder and ovarian (as confirmed here), have no recorded mutations to date.

**Table 3:** Somatic mutations identified in MAGE genes in genome wide surveys.

Gene	Sample name (tumor type)	Amino acid	Codon change	TP53 status	BRAF status	Reference
MAGEA1	HCC1008 (breast cancer cell line)	K278T	AAA>ACA	D281H	WT	[17]
MAGEA1	NCI-H1770 (NSCLC cell line)	A63P	GCC>CCC	R248W	WT	[22]
MAGEA4	HCC1008 (breast cancer cell line)	G153D	GGC>GAC	D281H	WT	[17]
MAGEA4	Br27P (glioma)	E221K	GAA>AAA	c.617delT (fs)	T310I	[18]
MAGEB6B	NCI-H2087 (NSCLC cell line)	G71>F	GGT>TTT	V157F	L597V	[22]
MAGEB10	Br09PT (glioma)	D55Y	GAT>TAT	W53X	WT	[18]
MAGEB10	LB647-SCLC (SCLC cell line)	Q148K	CAG>AAG	p.E294fs*51	WT	[22]
MAGEB16	NCI-H2009 (NSCLC cell line)	T302R	ACA>AGA	R273L	WT	[22]
MAGEB16	LB647-SCLC (SCLC cell line)	A279T	GCT>ACT	p.E294fs*51	WT	[22]
MAGEB16	HCC2218 (breast cancer cell line)	L323L	CTG>CTT	R283C	WT	[17]
MAGEC1	Br02X (glioma)	I1001F	ATT>TTT	WT	WT	[18]
MAGEC2	HCC1954 (breast cancer cell line)	G6C	GGC>TGC	Y163C	WT	[17]
MAGEC3	MZ7-mel (melanoma cell line)	D50N	GAC>AAC	WT	V600E	[22]
MAGEC3	CP66-MEL (melanoma cell line)	L551F	CTT>TTT	WT	WT	[22]
MAGED2	Hs 578T (breast cancer cell line)	K458Q	AAG>CAG	V157F	WT	[22]
MAGEE1	HCC2713 (breast cancer cell line)	Y640F	TAC>TTC	c.723delC (fs)	NA	[22]
MAGEE1	CP66-MEL (melanoma cell line)	R934K	AGG>AAG	WT	WT	[22]
MAGEE1	HCC1008 (breast cancer cell line)	T664N	ACC>AAC	D281H	WT	[17]
MAGEE1	Pa14C (pancreas tumor)	V649V	GTG>GTT	WT	WT	[19]
MAGEH1	Br23X (glioma)	A13A	GCG>GCA	WT	WT	[18]
MAGEH1	NCI-H2087 (NSCLC cell line)	F100F	TTC>TTT	V157F	L597V	[22]

*Two consecutive changes.

The recent sequencing of a melanoma genome has revealed a mutation spectrum reflective of the mutagenic impact of ultraviolet light [23]. This pattern can be clearly observed in this study in that C>T - G>A alterations represent almost 89% of the *MAGE* mutations found (Table S5), with over 90% of these occurring at dipyrimidine sites. This UV mutation signature pattern was also observed for *TP53* in the melanoma samples analyzed here. It was not, however, observed in the *MAGE* mutations found in other tumor types.

**Figure 1:**
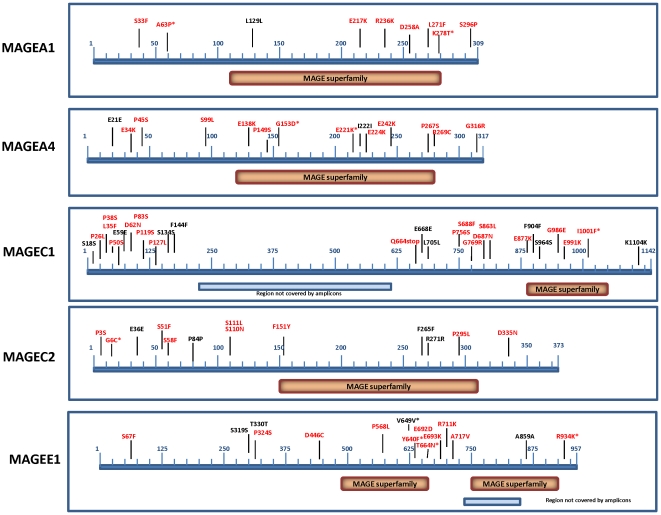
Distribution of mutations in the *MAGE* genes analyzed in this study. In red are the non-synonymous and in black the synonymous mutations. Asterisks indicate mutations that were identified in other studies.

Overall, the non-synonymous to synonymous ratio of the *MAGE* mutations was found to be 2.45∶1. This is not different from the ratio that would be expected to occur by chance. However, this low non-synonymous:synonymous ratio is largely due to a very high proportion of synonymous mutations in *MAGEC1* and *MAGEC2* (14 of 20). *MAGEA1*, *MAGEA4*, and *MAGEE1* have non-synonymous to synonymous ratios of 6∶1, 5∶1 and 2.66∶1 respectively. Moreover, all the mutations in the two *MAGEA* genes reported in the databases are non-synonymous (Table 1). Thus, *MAGEA* mutations might be drivers of tumorigenesis, as has previously been postulated for *MAGEE1*. Due to their distribution throughout the genes studied (Figure 1), we speculate that the *MAGE* mutations we have identified are more likely to be inactivating than activating. Consistent with this, for the *MAGEA* genes where the NS:S ratio is suggestive of their being drivers, there is evidence that both play tumor suppressive roles. *MAGEA1* expression was shown to correlate with good prognosis in neuroblastoma [14] and *MAGEA4* expression was shown to promote tumor cell death and sensitize lung malignancies to apoptotic stimuli, such as chemotherapeutic agents [16]. MAGEA4 was also shown to interact with gankyrin and to suppress its oncogenic activity [24]. It is thus possible, that the inactivation of *MAGEA1* and *MAGEA4*, which are generally expressed in coordination with other CT-X genes such as *MAGEA2* and *MAGEA3* for which there is evidence for oncogenic function, might enhance the overall tumorigenicity of coordinated CT-X expression leading to a net positive contribution to tumor progression.

Recently the X-ray structure of the MAGE homology domain of *MAGEA4* (PDB ID 2WA0) was determined. This permitted a more detailed analysis of the mutations that fall within this domain in the various family members, as described in Supplementary Methods S1. We found that the mutations found in this region involve residues that have a higher solvent exposure and a lower FoldX score [25] than average, implying that they do not play an important role in the structural integrity of the protein but might serve for interactions with other molecules (Supplementary Methods S1 and Table S6). Although it is unclear whether the MAGE homology domain is involved in binding interactions, this observation reinforces the possibility of the mutations resulting in discrete functional changes.

One other aspect of the MAGE gene mutations worthy of further investigation is their frequent occurrence in potential phosphorylation sites. As predicted by http://scansite.mit.edu/, these mutations could either abolish predicted existing sites or create new potential sites (A63P [new site at S62], K278T and S296P in MAGEA1; P45S, S99L, P149S and P267S in MAGEA4; P38S, P83S and S86L in MAGEC1; S51F, S58F, S110N, S111L, F151Y and P295L [new sites at S293, Y296 and Y297] in MAGEC2). Although at this stage we have no direct evidence that the MAGE proteins are phosphorylated, this observation does hint at potential functional consequence of many of the mutations.

Lastly, we considered the possibility that selective immune pressure that might underlie the *MAGE* mutations. In this context, six of the eight missense mutations identified in *MAGEA1* (residues 63, 236, 258, 271, 278, 296), the only one of the genes sequenced where extensive mapping of T cell epitopes has been performed, affect known epitopes [26]. Thus, the mutations might reduce antigenicity and serve as an alternate escape mechanism to loss of antigen or MHC expression. It remains to be determined, however, whether the mutated epitopes were involved in antigenicity in the patients where they were identified.

A notable facet of the *MAGE* mutations is their non-random distribution between patients. Thirty five of the fifty four *MAGE* mutations (64.8%) in our Validation and Discovery Screens are from samples that exhibit more than one mutation, often with multiple mutations in the same gene. For example, one sample in the Validation Screen, 4198, exhibited nine coding region mutations and another, 6985, six mutations. Even in the list of mutations identified in other studies, 41% are from samples where more than one *MAGE* mutation has been identified. These data suggest that a significant subset of the mutations might arise due to a DNA instability syndrome, either affecting the X-chromosome or the entire genome.

While our results do not as yet define the functional consequences of the MAGE gene mutations observed, they do demonstrate for the first time that this gene family is frequently mutated in melanoma. Therefore, our study argues for enhanced efforts to discern potential tumorigenic properties of these genes that serve as the platform for therapeutic cancer vaccines already in advanced clinical development.

## Supporting Information

Supplementary Methods S1 Homology Models of the MAGE Homology Domain
(0.05 MB DOC)

Table S1 Melanoma cell lines used in the Discovery Screen
(0.03 MB XLS)

Table S2 Clinical characteristics of the Melanoma patients included in the Validation Screen
(0.13 MB XLS)

Table S3 Primer sequences used in this study
(0.06 MB XLS)

Table S4 Somatic TP53 mutations identified in ovarian tumors
(0.03 MB DOC)

Table S5 Spectrum of the MAGE mutations in the discovery and validation sets
(0.04 MB DOC)

Table S6 Mutations in MAGE proteins, FoldX score, solvent accessible surface area and conservation score.
(0.04 MB DOC)
