# Supplementary material for: Frequent MAGE Mutations in Human Melanoma
Source: PLoS One. 2010 Sep 16;5(9):e12773. doi: 10.1371/journal.pone.0012773 (PMC2940856; doi:10.1371/journal.pone.0012773)
Supplement: Supplementary Methods S1 — Homology Models of the MAGE Homology Domain (0.05 MB DOC) [file pone.0012773.s001.doc]

**Methods S1**

**Homology Models of the MAGE Homology Domain**

Residues 102–312 of the recently resolved structure of human MAGE A4 (PDB ID 2WA0) comprise the full MAGE domain (residues 117–287) and served as a template for all structural models. The missing residues 172–173, 192–193, and 263–268 were added to the X-ray structure using the MODELLER program [1], producing the reference structure for all MAGE protein models.

Multiple sequence alignments for human, chimpanzee, and mouse MAGE proteins were generated. Human MAGEs have been manually collected. Chimpanzee MAGEs were predicted using profiles derived from the human MAGE sequences. Mouse MAGE sequences were predicted using profiles derived from a combination of human and chimpanzee sequences. The data set contained 21 MAGE A sequences, 20 MAGE B sequences and 7 MAGE C sequences. These were used to build models with the MODELLER program. 500 models were calculated for each sequence and scored with the ANOLEA [2] estimate of the folding free energy for each amino acid, using the default 5 residues window averaging. The clustering routine from the MMTSB Tool Set [3] was used to cluster the models, and the model with the best ANOLEA energy from the largest cluster was used for further refinement by minimization with the CHARMM program [4] and the CHARMM22 all atom force field [5]. Minimization consisted of 100 steps of steepest descent, with a harmonic restraint of 5 kcal/mol/Å^2^ imposed on all heavy atoms towards their initial position.

To estimate the role played by each residue for the protein stability, the FoldX program [6] was used to perform an *in-silico* alanine scan by mutating each residue to an alanine and estimating the change of folding energy. The solvent accessible surface area for each residue was calculated with CHARMM using a probe radius of 1.4 Å. The conservation score for each amino acid residue as calculated using SCORECONS (method: valdar01) with default parameters [7].

**References**

1. Sali A, Blundell TL (1993) Comparative protein modelling by satisfaction of spatial restraints. J Mol Biol 234: 779-815.

2. Melo F, Feytmans E (1997) Novel knowledge-based mean force potential at atomic level. J Mol Biol 267: 207-222.

3. Feig M, Karanicolas J, Brooks CL, 3rd (2004) MMTSB Tool Set: enhanced sampling and multiscale modeling methods for applications in structural biology. J Mol Graph Model 22: 377-395.

4. Brooks BR, Bruccoleri RE, Olafson BD, States DJ, Swaminathan S, et al. (1983) CHARMM: A program for macromolecular energy, minimization, and dynamics calculations. Journal of Computational Chemistry 4: 187-217.

5. MacKerell AD, Bashford D, Bellott, Dunbrack RL, Evanseck JD, et al. (1998) All-Atom Empirical Potential for Molecular Modeling and Dynamics Studies of Proteinsâ€ The Journal of Physical Chemistry B 102: 3586-3616.

6. Guerois R, Nielsen JE, Serrano L (2002) Predicting changes in the stability of proteins and protein complexes: a study of more than 1000 mutations. J Mol Biol 320: 369-387.

7. Valdar WS (2002) Scoring residue conservation. Proteins 48: 227-241.
