# Supplementary material for: Frequent MAGE Mutations in Human Melanoma
Source: PLoS One. 2010 Sep 16;5(9):e12773. doi: 10.1371/journal.pone.0012773 (PMC2940856; doi:10.1371/journal.pone.0012773)
Supplement: Table S4 — Somatic TP53 mutations identified in ovarian tumors (0.03 MB DOC) [file pone.0012773.s005.doc]

**Table S4**. Somatic TP53 mutations identified in ovarian tumors

| **Sample** | **Amino acid position** |
| --- | --- |
| TU0412255MDA | R273C |
| TU13709MDA | R273C |
| TU1278009MDA | G266V |
| TU1286604MDA | R280stop |
| TU0412302MDA | R273H |
| TU14034MDA | V272M |
| TU14283MDA | R282W |
| TU1286537MDA | A161T |
| TU14333MDA | S127F |
