# Supplementary material for: Frequent MAGE Mutations in Human Melanoma
Source: PLoS One. 2010 Sep 16;5(9):e12773. doi: 10.1371/journal.pone.0012773 (PMC2940856; doi:10.1371/journal.pone.0012773)
Supplement: Table S5 — Spectrum of the MAGE mutations in the discovery and validation sets (0.04 MB DOC) [file pone.0012773.s006.doc]

**Table S5: Spectrum of the *MAGE* mutations in the discovery and validation sets**

| **Mutation** | **All genes** | ***MAGEA1*** | ***MAGEA4*** | ***MAGEC1*** | ***MAGEC2*** | ***MAGEE1*** |
| --- | --- | --- | --- | --- | --- | --- |
| C>T | 37 | 2 | 6 | 17 | 6 | 6 |
| G>A | 25 | 3 | 6 | 9 | 5 | 2 |
| A>G | 1 | - |  | - | - | 1 |
| C>G | 1 | - | - | - | - | 1 |
| T>C | 1 | 1 | - | - | - | - |
| A>C | 1 | 1 | - | - | - | - |
| A>T | 1 | - | - | - | - | 1 |
| C>A | 1 | - | - | 1 | - | - |
| G>C | - | - | - | - | - | - |
| G>T | 1 | - | - | - | - | 1 |
| T>A | 1 | - | - | - | 1 | - |
| T>G | - | - | - | - | - | - |
