# Supplementary material for: Frequent MAGE Mutations in Human Melanoma
Source: PLoS One. 2010 Sep 16;5(9):e12773. doi: 10.1371/journal.pone.0012773 (PMC2940856; doi:10.1371/journal.pone.0012773)
Supplement: Table S6 — Mutations in MAGE proteins, FoldX score, solvent accessible surface area and conservation score. (0.04 MB DOC) [file pone.0012773.s007.doc]

**Table S6:** Mutations in MAGE proteins^&^, FoldX score (FoldX, [kcal/mol]), solvent accessible surface area (SASA, [Å^2^]), conservation score.

| Mutation | FoldX* | SASA | Conservation score |
| --- | --- | --- | --- |
|  | MAGEA1 (94-304) | | |
| E217K | -0.72 | 82.61 | 0.39 |
| R236K | **5.52** | 80.07 | 0.84 |
| D258A | -0.75 | 55.53 | 0.79 |
| L271F | 0.93 | 101.34 | 0.53 |
| K278T | 0.84 | 83.26 | 0.84 |
| S296P | 1.15 | 38.43 | 0.43 |
|  | MAGEA4 (102-312) | | |
| E138K | 0.44 | 105.62 | 0.52 |
| P149S | **2.21** | 91.06 | 0.85 |
| G153D | -1.43 | 20.04 | 0.46 |
| E221K | 0.16 | 60.94 | 0.47 |
| E224K | -1.10 | 128.03 | 0.84 |
| E242K | **3.00** | 46.53 | 0.72 |
| P267S | 0.61 | 126.24 | **1.00** |
| R269C | 1.28 | 145.46 | 0.59 |
|  | MAGEB10 (103-313) | | |
| Q148K | -0.21 | 0.23 | 0.58 |
|  | MAGEC1 (900-1109) | | |
| G986E | 1.82 | 7.41 | 0.75 |
| E991K | -2.27 | 33.48 | 0.48 |
| I1001F | 2.16 | 10.63 | 0.75 |
|  | MAGEC2 (133-339) | | |
| E143K | -0.94 | 102.15 | 0.44 |
| F151Y | **3.34** | 22.28 | **0.90** |
| F178V | **3.49** | 29.13 | **0.96** |
| P295L | 1.83 | 97.36 | 0.67 |
| D335N | -3.56 | 17.12 | 0.54 |

^&^Include mutations from the Discovery and Validation Screens in this study, as well as mutations found in the Genome Wide mutation surveys.

^#^ conservation score as calculated with the program SCORECONS.

*Residues with a high FoldX score (> 2 kcal/mol) that may therefore be important for protein structure, are marked in bold.
